# Transmission of Infectious Diseases En Route to Habitat Hotspots

**DOI:** 10.1371/journal.pone.0031290

**Published:** 2012-02-20

**Authors:** Julio Benavides, Peter D. Walsh, Lauren Ancel Meyers, Michel Raymond, Damien Caillaud

**Affiliations:** 1 CNRS-Institut des Sciences de l'Evolution de Montpellier, Université de Montpellier II, Montpellier, France; 2 Department of Archaeology and Anthropology, University of Cambridge, Cambridge, United Kingdom; 3 Section of Integrative Biology, The University of Texas at Austin, Austin, Texas, United States of America; 4 Division of Statistics and Scientific Computation, The University of Texas at Austin, Austin, Texas, United States of America; 5 Santa Fe Institute, Santa Fe, New Mexico, United States of America; University of California, Berkeley, United States of America

## Abstract

**Background:**

The spread of infectious diseases in wildlife populations is influenced by patterns of between-host contacts. Habitat “hotspots” - places attracting a large numbers of individuals or social groups - can significantly alter contact patterns and, hence, disease propagation. Research on the importance of habitat hotspots in wildlife epidemiology has primarily focused on how inter-individual contacts occurring at the hotspot itself increase disease transmission. However, in territorial animals, epidemiologically important contacts may primarily occur as animals cross through territories of conspecifics en route to habitat hotspots. So far, the phenomenon has received little attention. Here, we investigate the importance of these contacts in the case where infectious individuals keep visiting the hotspots and in the case where these individuals are not able to travel to the hotspot any more.

**Methodology and Principal Findings:**

We developed a simulation epidemiological model to investigate both cases in a scenario when transmission at the hotspot does not occur. We find that (i) hotspots still exacerbate epidemics, (ii) when infectious individuals do not travel to the hotspot, the most vulnerable individuals are those residing at intermediate distances from the hotspot rather than nearby, and (iii) the epidemiological vulnerability of a population is the highest when the number of hotspots is intermediate.

**Conclusions and Significance:**

By altering animal movements in their vicinity, habitat hotspots can thus strongly increase the spread of infectious diseases, even when disease transmission does not occur at the hotspot itself. Interestingly, when animals only visit the nearest hotspot, creating additional artificial hotspots, rather than reducing their number, may be an efficient disease control measure.

## Introduction

The spread of infectious diseases strongly depends on how habitat characteristics shape patterns of between-host interactions [1], [2]. In particular, habitat heterogeneity influences patterns of between-individual contacts and hence, disease dynamics [1], [3]. For example, “habitat hotspots”, sites that attract individuals or social groups over long distances, can be visited by a large subset of a population. Around hotspots, between-individual contact rates often increase in frequency, which amplifies disease transmission. In humans, schools and working places are typical examples of hotspots and have been shown to accelerate the spread of measles, influenza and SARS [4], [5], [6]. Thus, limiting transmission at hotspots has become a promising strategy for mitigating epidemics (*e.g.*, influenza [7]) although the efficiency of such strategies also depends on the role hotspots plays relative to other sources of local transmission (*e.g.*, influenza [6], [7])

In wild animal populations, high quality feeding spots (*e.g.*, fruit trees), breeding sites, waterholes or sleeping sites can exacerbate direct physical contacts. Empirical and theoretical studies on the epidemiological importance of habitat hotspots have mainly focused on how the spatial aggregation of animals favors disease transmission at the hotspot itself [8], [9]. For example, the aggregation of wild boar at watering sites significantly increases the transmission of tuberculosis-like lesions [8]. However, inter-individual contacts may not always significantly increase at the hotspot itself. This is for example the case of habitat hotspots that some animal species only visited occasionally, such as some mineral licks [10], [11]. Also, animals present at the same time at a particularly large hotspot may not be close enough to each other to transmit infectious diseases. This is the case of large forest clearings [12], [13] or large waterholes. Finally, species such as primates and ungulates might avoid defecating in hotspots of high food resources, limiting the transmission of fecal-oral parasites at hotspots [14], [15].

When disease transmission does not occur at the hotspot, it can still occur at a certain distance from the hotspot. This phenomenon has received little attention so far. Specifically, infective contacts may be observed when infectious individuals travel to the hotspot and cross the territory of susceptible individuals and, reversely, when susceptible individuals cross the territory of infectious individuals. This second type of transmission may be prominent when the disease reduces the mobility of sick individuals (*i.e.*, sickness behavior [16], [17], [18]). For example, in humans, sick individuals often stay home, which alters disease dynamics [19], [20]. Sick wild animals also commonly reduce their rate of search for food or water [21]. Such transmission may particularly apply to parasites that can survive in the environment (*e.g.*, gastrointestinal parasites) for which the spatial overlap of the home ranges of sympatric hosts favors transmission [22].

To investigate these transmission mechanisms, we developed an agent-based model exploring patterns of disease spread in a large closed population composed of territorial social groups, in which one or more hotspots influence group movement patterns, but where direct disease transmission at the hotspot itself is negligible. Our hypothesis is that terrestrial animals necessarily cross conspecifics' home ranges on their way to a hotspot, which modifies the contact network of the population and may subsequently alter disease transmission. We assumed that between-group disease transmission can occur both between groups having neighbouring territories and between groups travelling to a hotspot and groups whose territories are crossed en route. We also assumed that only groups which territory lies within a certain distance from the hotspot (further referred as “radius of attraction”) can visit it, and that their visitation rate decreases as this distance increases.

The relationship between the radius of attraction and the disease dynamics was then investigated under two scenarios: i) when groups including sick individuals do not travel to the hotspot, and ii) when these groups still travel to the hotspot. The first scenario corresponds to the case of virulent parasites that can strongly decrease the mobility of infected individuals, such as Ebola virus in western lowland gorillas [23], whereas the second scenario applies to pathogens that do not strongly modify the behavior of their host, such as some gastro-intestinal macro-parasites and bacteria [24]. Under both scenarios, we investigated the relationship between the disease attack rate and the hotspot radius of attraction, identified the groups in the population that have the highest risk of infection and explored the relationship between the number of hotspots and the magnitude of an epidemic.

## Methods

### General characteristics

**Figure 1:**
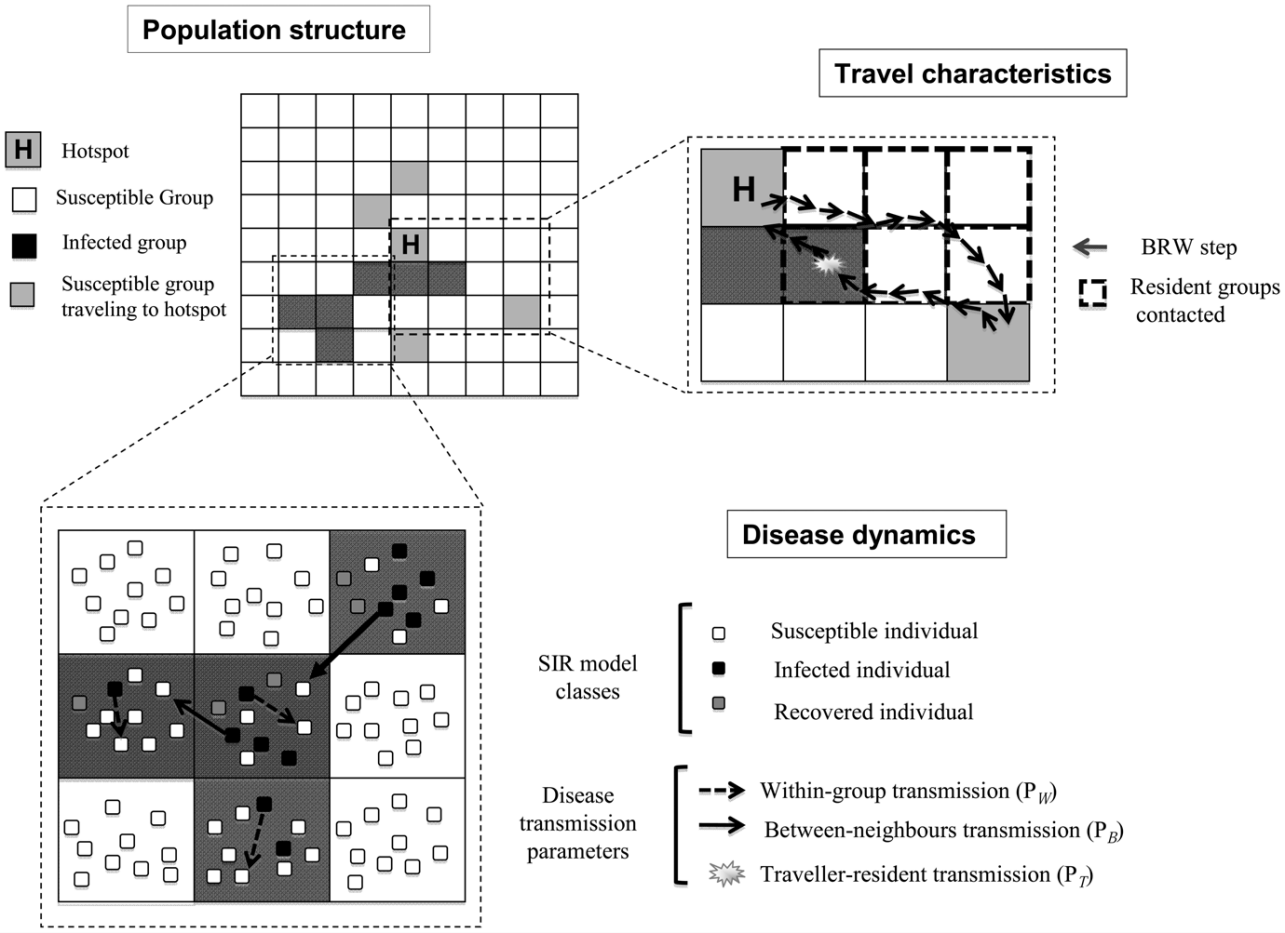
Model schematic. The hotspot is located at the center of a 51×51 lattice. All other cells correspond to a group's territory. Groups with at least one infected individual are considered infected, indicated in dark grey. A 9×9 section of the lattice depicts the SIR transmission dynamics among individuals that are either in the same group or neighbouring groups (bottom). Groups follow Biased Random Walks (BRW) during their daylong trips to the hotspot (top right). Transmission is possible between a travelling group and the groups residing in cells traversed en route to the hotspot.

The model has a 51×51 lattice structure, where each cell of the lattice corresponds to a group's territory. We assumed disease transmission can occur between each group and its eight neighbours (Fig. 1). We use *N_i_* to denote the list of indices of the eight neighbours of group *i*. Initially, a single habitat hotspot is placed at the center of the lattice. All groups are assumed to include ten individuals. At each daily time step, each group either visits the hotspot or stays in its territory. The probability 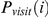 of a visit by group *i* is a decreasing function of the Euclidean distance, 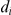, between the group's territory and the hotspot. We assume that all groups gain the same benefit from visiting the hotspot and that the travel cost is proportional to 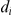, leading to:
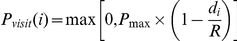
where *P_max_* is the probability of a visit for the eight groups directly neighbouring the hotspot, and *R* is the hotspot radius of attraction. Groups occupying cells that are farther from the hotspot than *R* never visit it.

When a group visits the hotspot, it follows a Biased Random Walk from its home cell to the hotspot (BRW [25]) and returns to its home cell on the same day. The length of each step of the BRW (denoted *S*) is held constant and the direction of the step is consistently biased towards the hotspot during the approach to the hotspot and towards the group's home cell during the return from the hotspot. Each turning angle is randomly drawn from a normal distribution N (0, *σ^2^*), where *σ* is a standard deviation parameter. The list of groups residing in cells encountered along each BRW to the hotspot is recorded. Groups travel to the hotspot and come back within a single time step.

At each time step, each group interacts with (i) groups occupying neighbouring cells (neighbour-neighbour contact), and (ii) if the group travels to the hotspot, all of the groups it encounters along the BRW (traveler-resident contact).

### Disease dynamics

We model infectious disease dynamics using a simple stochastic susceptible-infectious-removed (SIR) epidemic model, with one-day time steps. Each individual moves independently through the three states: Susceptible (at risk of contracting the disease), Infectious (capable of transmitting the disease), or Removed (recovered or dead). Susceptible individuals can be infected by infected individuals from either its own group or other groups. The latter can occur during neighbour-neighbour contacts or traveler-resident contacts.

The local transmission probability *P_i_* is defined as the per-time-step probability that a susceptible individual in group *i* is infected by an infectious individual from its own group or a neighbouring group. Let *I_i_* denote the number of infectious individuals in group *i*. The probability that the focal individual is infected by at least one of the infectious individuals in its own group is 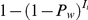, where 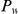 is the within-group transmission probability from an infected individual to a susceptible individual of the same group. Likewise, the probability of a susceptible individual being infected by an infectious individual from one of the eight neighbouring groups is 
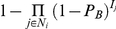
, where *P_B_* is the between-group transmission probability from an infected individual to a susceptible individual of a neighbouring group. Combining these two sources of infection, the local transmission probability is given by
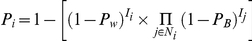



The per-time-step probability of becoming infected during transit to a hotspot 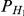 depends on the number of infectious individuals *I_c_* in each group *c* encountered en route and the *P_T_* “travelling” probability of transmission during one of these transient contacts between a resident and traveling group. Specifically, during a one-day trip to a hotspot, the probability that a susceptible individual in the group is infected along the way is given by
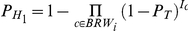
where 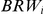 denotes the list of indices of the groups encountered by group *i* as it travels to and from the hotspot.

In a second version of the model, infected groups are assumed to travel to the hotspot. Transmission from infected travelers to susceptible residents encountered en route can then occur. In this case, the per day probability that a susceptible individual in group *i* is infected by a traveler depends on the numbers of infected individuals in each of the groups that travels through the territory of *i* en route to the hotspot, and is given by
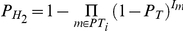
where 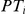 denotes the set of groups passing through *i*'s territory.

At the end of each time step, each infected individual is removed with probability *γ*. We assume that no transmission occurs between groups travelling to the hotspot simultaneously.

### Model versions

We explored two versions of the transmission model. In the “Sick-stay” model, groups that included at least one infected individual – infected groups – were assumed to stop travelling to the hotspot; in the “Sick-travel” model, infected groups continue to travel to the hotspot as if uninfected. In the Sick-stay model, disease transmission between travelers and residents can only occur from an infected resident to a susceptible traveler, while in the Sick-travel model, transmission can be bi-directional.

We also considered models with multiple hotspots. A specified number of hotspots are randomly placed on the lattice, and groups visit only their nearest hotspot (according to *P_visit_*, described above).

### Initial conditions

At the beginning of each simulation, all individuals were susceptible. Epidemics were started with a single infected individual. Unless stated otherwise, the first case was introduced into one of the eight groups adjacent to the hotspot. At each time step, the number of infected and removed individuals (and groups) was recorded until no individual in the population was infected, which indicated the end of the epidemic. For each parameter combination, we ran 1000 simulations.

For all simulations, the recovery rate *γ* was set to 0.1, the maximum probability of a visit to the hotspot, *P_max_*, was set to 0.1, and the BRW step length *S* was set to 0.25 (*i.e.*, 1/4 of the distance between the center of neigbouring group's territories). We also assumed that the within-group transmission probability, *P_w_*, was at least ten times higher than the between-group transmission probabilities (*P_B_* and *P_T_*).

The model was implemented in Delphi 7 (Borland Software Corporation, 2002). Table 1 summarizes parameter definition and values, and a sample run of the model is shown in Video S1.

**Table 1:** Overview of processes and parameters of the model.

Parameter	Value
**Population structure**	
Number of groups	2601
Group size	10
**Hotspot travel characteristics**	
*R*: Hotspot radius of attraction	0–35
*P_max_*: Visit probability for groups located next to the hotspot	0.1
*S*: Step length of the Biased Random Walk (BRW)	0.25
*σ*: Standard deviation for the BRW deviation angle	2
**Disease dynamics**	
*γ*: Recovery rate (days^−1^)	0.1
*P_w_*: Within-group transmission probability	0.02–0.06
*P_B_*: Between-neighbours transmission probability	4e-04 – 16e-04
*P_T_*: Traveler-resident transmission probability	1e-06 – 1e-03

## Results

### Epidemiological impacts of transmission rates and location of first case

**Figure 2:**
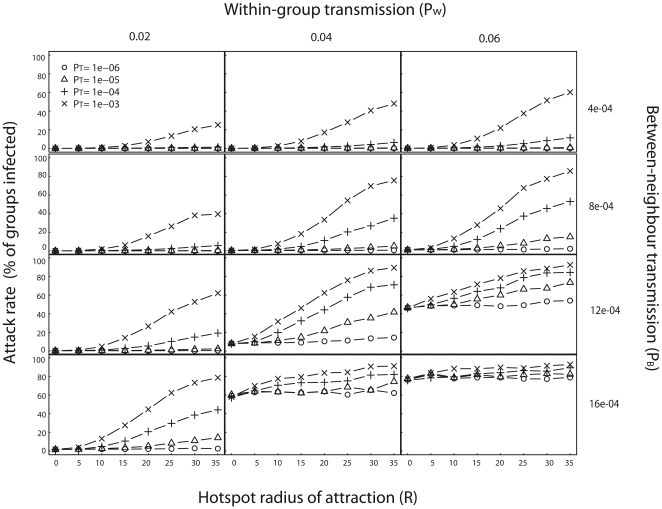
Influence of multiple model parameters on attack rate, when infected groups do not travel (Sick-stay model). The fraction of groups infected increases with the hotspot radius of attraction, but varies with the traveler-resident transmission probability *P_T_* (four lines in each graph), within-group transmission probability *P_w_* (three different columns of graphs), and between-neighbour transmission probability *P_B_* (four different rows of graphs). Each value is based on 1000 simulations in which disease was introduced randomly in one of the eigth groups adjacent to the hotspot.

The attack rate (proportion of groups becoming infected following a single disease introduction) generally increases with the hotspot radius of attraction *R*, and the traveler-resident transmission probability *P_T_* (Fig. 2). This occurs whether or not infected groups are assumed to travel during infection (Fig. 2 and Fig. S1 in supplemental materials). As predicted by percolation theory [26], the attack rate also increases with both the within-group transmission parameter *P_W_* and the between-group transmission parameter *P_B_*. Interestingly, the highest impact of the hotspot radius of attraction on the attack rate was observed for intermediate values of *P_W_* and *P_B_*. For low values of *P_W_* and *P_B_*, inter-group disease transmission primarily occurred between travelling and resident groups and was often not sufficient to sustain an epidemic. For high values of *P_W_* and *P_B_*, the disease always percolated, even in the absence of the hotspot. For intermediate values of *P_W_* and *P_B_*, groups infected en route to the hotspot then stochastically triggered small outbreaks around their territories. The epidemiological impact of the hotspot was amplified by this interaction between traveler-resident transmission and neighbour-neighbour transmission.

**Figure 3:**
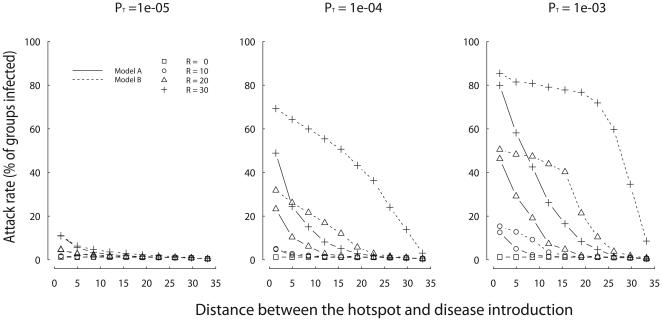
Attack rate decreases with the distance between the hotspot and point of disease introduction. Sick-stay model (solid lines) and Sick-travel model (dashed lines) are compared for different values of the hotspot radius of attraction (*R*). Each graph presents a different value of the traveler-resident transmission rate (*P_T_*). Each value is averaged over 1000 stochastic simulations, assuming *P_B_* = 8e-04 and *P_w_* = 0.06.

In both models, the attack rate decreased as the distance between the hotspot and the point of disease introduction increased (Fig. 3). The greater this distance, the lower the probability that a group visiting the hotspot encountered the group initially infected. The Sick-travel model yields higher attack rates than the Sick-stay model, particularly for groups ranging at intermediate distances between the location of the first disease case and the hotspot.

### Group-specific epidemiological risk

**Figure 4:**
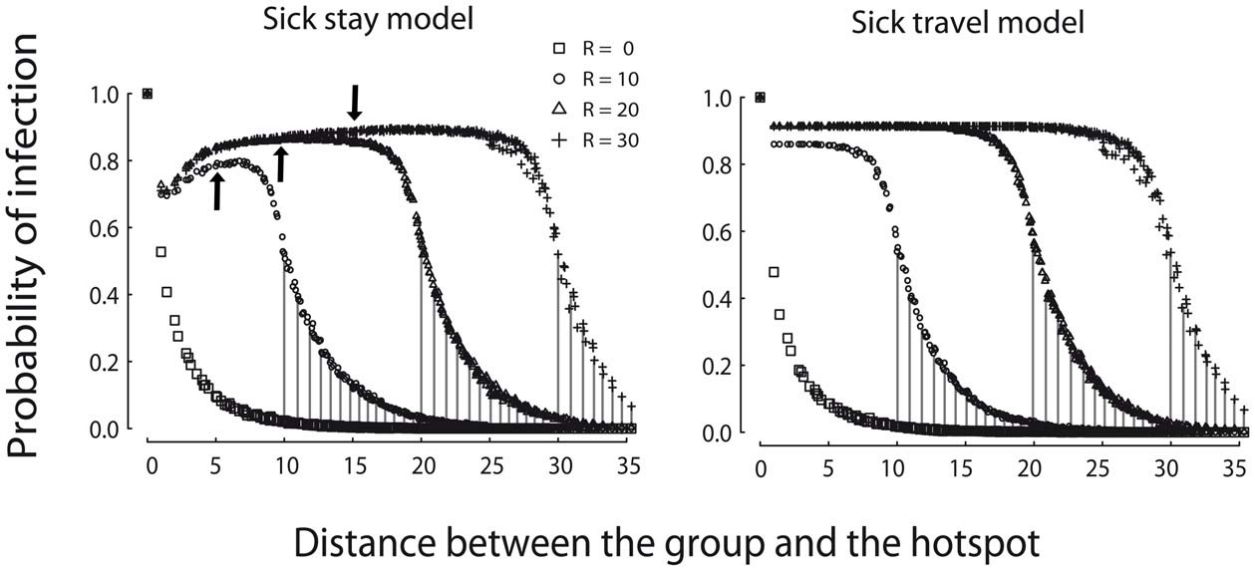
Probability of infection depends on distance to hotspot. The relationship is presented for different values of the hotspot radius of attraction (*R*) in Sick-stay model (left) and Sick-travel model (right). Vertical lines compare the probability of infection when there is no hotspot (*R* = 0) to the probability of infection when there is a hotspot (*R*>0), for groups residing beyond the radius of attraction (distance to hotspot greater than *R*). This quantifies the indirect epidemiological impact of the hotpot on groups that never travel themselves or encounter travelers en route to the hotspot. Black arrows show the analytical prediction of the most vulnerable group to disease for R = 10, 20 and 30 respectively. Parameter values are *P_B_* = 8e-04, *P_T_* = 0.001 and *P_w_* = 0.06. Each value is based on 1000 stochastic simulations in which disease was introduced randomly in one of the eight groups adjacent to the hotspot. Results for other parameter values are shown in Fig. S2 and S3.

In the Sick-travel model, groups ranging closer to the hotspot exhibited higher probabilities of infection (Fig. 4 and Fig. S2 in supplemental materials), since their territories are crossed by large numbers of infected groups travelling to the hotspot (Fig. S4a). The relationship is more complex in the Sick-stay model: groups ranging at intermediate distances from the hotspot experience the highest risks of infection (Fig. 4 and Fig. S3 in supplemental materials). In this case, hotspot-mediated infection occurs only from infected residents to susceptible travelers. Groups ranging close to the hotspot travelled more often, but encountered only a small number of potentially infected groups. Groups ranging far from the hotspot encountered larger numbers of groups when visiting the hotspot, but did so only rarely. Thus groups ranging at intermediate distances experienced the greatest number of potentially infective contacts with resident groups encountered en route to the hotspot (Fig. S4b). When epidemics occur, the difference in spatial pattern of disease spread observed between the models is insensitive to the parameter values (Fig. S2 and Fig. S3).

The fact that groups ranging at an intermediate distance from the hotspot display a higher number of potentially infective contacts can easily be understood using a simple mathematical model. Indeed, the expected number of groups encountered by a group *i* visiting the hotspot, per time unit, can be assumed to be approximately proportional to *P_visit_* and to the territory-hotspot distance *d_i_*:
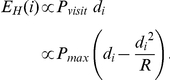
This approximation holds as long as *d_i_* is large or the turning angle is low. The derivative of this second-order polynomial has a maximum in 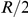.

Second, we assessed the epidemiological impact of the hotspot on groups that never visit it because they range at a distance larger than *R* from the hotspot. For both models, when *P_B_* is high enough to allow some between-neighbour transmission, the attack rate for these groups was found to be larger than expected under a model with no hotspot (*R* = 0) (Fig. 4). Stochastic, local between-group transmission events allow the spread of the disease beyond the radius of attraction. Groups that never visit the hotspot are thus indirectly impacted by the hotspot.

Finally, outside of the radius of attraction, disease spreads as expected for a lattice model [27] whereas inside the radius of attraction, disease spread rapidly among the groups, with no apparent spatial structure.

### Number of hotspots

**Figure 5:**
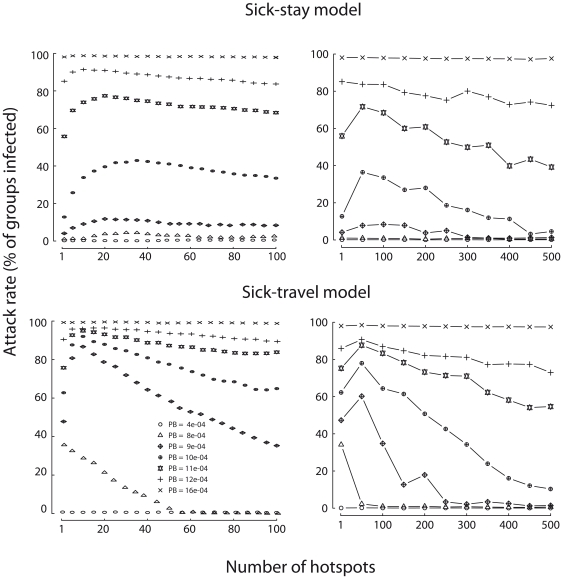
Number of hotspots. Each line graphs the change in attack rate as a function of the number of hotspots, for a different value of *P_B_* (from 4e-04 to 16e-04). Results are presented for hotspots ranging from 1–100 (left) and 1–500 (right) in the Sick-stay model (top) and the Sick-travel model (bottom). Each value is averaged over 1000 stochastic simulations assuming *R* = 30, *P_w_* = 0.06, *P_T_* = 4e-04. Each hotspot was located randomly in the population, and disease was introduced into the group ranging in the middle of the habitat.

For both models, the relationship between the number of hotspots and the attack rate is bell-shaped (Fig. 5). For a low number of hotspots, adding new hotspots increases the fraction of the population ranging within the radius of attraction of these hotspots and, thereby, increases the overall attack rate. Beyond a certain number of hotspots, however, all groups are already attracted by at least one hotspot on the landscape. Under the assumption that these groups travel exclusively to the nearest hotspot, adding more hotspots decreases the distance travelled by the groups and the number of infective contacts they can have en route, and thereby lowers the attack rate.

## Discussion

Spatial features of the landscape such as habitat hotspots can profoundly influence the spread of infectious diseases [28], [29]. Our model extends previous studies focusing on transmission at the hotspot, and reveals that hotspots can also strongly alter disease transmission by generating infective contacts between animals travelling towards or from the hotspot and animals whose territories are traversed. Our results show that even when sick groups stay in their territory, hotspots may increase the size of an epidemic. When infected animals cease to visit the hotspot, groups ranging at intermediate distances to the hotspot are the most vulnerable. We also found that the epidemiological impact of hotspots extends far beyond the subset of the population that visits it; even groups having no contact with those visiting the hotspot display elevated risks of infection. Finally, our model predicts that when groups visit their nearest hotspots, the epidemiological impact of hotspots is most severe when the number of hotspots is intermediate.

Hotspots impact disease transmission via a combination of both local between-neighbour and long-range traveler-resident transmissions, which is characteristic of a small-world network [30]. Disease dynamics in our model resemble those in a classic small-world network in several aspects. First, the attack rate increases with long-distance interactions, determined by the hotspot radius of attraction (Fig. 2). Second, new foci of infection established by long-distance traveler-resident contacts only spread when the local transmission rate, between neighbours, is sufficiently high. This phenomenon extends the influence of the hotspot beyond the radius of attraction (Fig. 4). Finally, as in small-world networks [31], all groups within the hotspot radius of attraction were infected almost at the same time. Thus, habitat hotspots potentially play a significant role in fuelling disease outbreaks, much like other natural mechanisms that generate small-world networks, such as the movement of vectors between plants [32], [33].

We find that hotspots are expected to influence disease dynamics significantly, even when infected groups do not travel to the hotspot at all. However, in this case, the hotspot effect strongly decreases as the distance between disease introduction and the hotspot increases. The reduction of mobility in infected groups also generates an unexpected spatio-temporal pattern: groups ranging at intermediate distance from the hotspot have the highest risk of infection, even if the disease is introduced immediately next to the hotspot. This counterintuitive result highlights the importance of understanding the behavioral effects of disease in wild animal populations. For example, as in humans, predicting the impact of hotspots on disease dynamics will strongly depend on understanding whether infectious individuals still travel to hotspots because disease symptoms appear after an infectious state (*e.g.*, influenza H1N1 [6], [19]), or whether infectious individuals do not visit hotspots because disease symptoms appear before the infectious state (*e.g.*, SARS [34], [35]). Furthermore, our results suggest that when transmission does not directly occur at hotspots, disease control measures targeting groups residing around the hotspot might not necessarily be the most efficient ones. Further simulation work is needed to identify optimal disease control measures.

The habitat of wild animal populations often includes more than one hotspot. For example, the habitat of terrestrial mammals can include a small number of high-value hotspots attracting dozens of groups (*e.g.*, salt licks or forest clearings) and more numerous low-value hotspots attracting only a few groups (*e.g.*, fruiting trees). Our model reveals that, when groups are assumed to travel to their nearest hotspot, the impact of disease outbreaks is a bell-shaped function of the number of hotspots (Fig. 5). This result challenges the hypothesis that the number of hotspots and disease prevalence will correlate positively [8], and could be used to optimize strategies for controlling disease in wild animal populations. Thus, wildlife managers may consider increasing, rather than decreasing [36], the number of water holes in order to reduce the number of highly-connected individuals or social groups, and hence the impact of an outbreak. However, additional studies are needed to determine if our result still holds when each animal visits more than one hotspot.

The values of the parameters of our model can be estimated from empirical data. The relationship between the distance from a group's territory and the hotspot visitation rate can be estimated using capture-mark recapture and telemetric data, between-group contact rates can be estimated from direct observation or telemetric data, and plausible distributions of disease transmission rates can be found in the literature. The step length of the biased random walk is assumed to have a fixed value (here, 0.25 times the size of a territory). This parameter does not need to be estimated accurately since it is redundant with another parameter, the traveler-resident contact rate, which is allowed to vary. Thus, the model can be applied to a broad range of host-parasite systems, from primate groups travelling to waterholes on a daily basis [37], [38] to large mammals visiting every few weeks mineral-rich areas [12], [13], [39]. In our model, the impact of the hotspot is particularly sensitive to the ratio between the local and the traveler-resident between-group transmissions. When the local between-neighbour transmission is high compared to the traveler-resident transmission, the impact of the hotspot is minimal.

We considered two discrete transmission scenarios, the Sick-travel and the Sick-stay scenarios. However, intermediate scenarios are also possible. For example, infected groups may fission such that only healthy individuals travel to the hotspot. In this case, we expect that although the overall disease transmission will increase compared to the pure Sick-stay scenario, the spatial pattern of the disease impact will be qualitatively similar to that observed for the Sick-stay model.

In this study, we have shown how transmission occurring around habitat hotspots influences disease transmission patterns, while previous studies have focused on disease transmission occurring at the hotspot itself. In some ecological systems, both transmission modes may coexist. For example, some fecal-orally transmitted parasites can infect both the soil and waterholes, and spore-forming bacteria such as *Bacillus anthracis* can persist for extended periods of time in animal carcasses, water and soil [40]. Additional works are needed to understand such epidemiological systems.

## Supporting Information

Figure S1 **Influence of multiple model parameters on attack rate, when infected groups travel (Sick-travel model).** The fraction of groups infected increases with the hotspot radius of attraction, but varies with the traveler-resident transmission probability *P_T_* (four lines in each graph), within-group transmission probability *P_w_* (three different columns of graphs), and between-neighbour transmission probability *P_B_* (four different rows of graphs). Each value is based on 1000 simulations in which disease was introduced randomly in one of the eigth groups adjacent to the hotspot.
(TIF)

Figure S2 **Group's probability of infection in relation to the distance to the hotspot, predicted by the Sick-travel model.** The relationship is presented for different values of the hotspot radius of attraction (*R*). Each graph represents a combination of the between-neighbour (*P_B_*) and the traveler-resident (*P_T_*) transmission. The disease was introduced randomly in one of the eight groups adjacent to the hotspot. For all simulations, *P_w_* = 0.06.
(TIF)

Figure S3 **Group's probability of infection in relation to the distance to the hotspot, predicted by the Sick-stay model.** The relationship is presented for different values of the hotspot radius of attraction (*R*). Each graph represents a combination of the between-neighbour (*P_B_*) and the traveler-resident (*P_T_*) transmission. The disease was introduced randomly in one of the eight groups adjacent to the hotspot. For all simulations, *P_w_* = 0.06.
(TIF)

Figure S4 **Traveler-resident contact patterns.** Each graph shows the relationship between the distance of a group from the hotspot and (a) the number of other groups that travel through its territory when travelling to and from the hotspot, (b) the number of resident groups it encounters when travelling to and from the hotspot. Values are based on encounters occurring during 100 time steps, in the absence of disease transmission.
(TIF)

Video S1 **Model dynamics.** The model shows one simulation run corresponding to the Sick-travel model. White, red and black squares represent susceptible, infected and removed groups, respectively. Blue squares represent groups travelling to the hotspot at each time step. The hotspot, in green, is in the middle of the lattice. The disease is introduced at the periphery.
(WMV)
