# Supplementary figures and images for: Transmission of Infectious Diseases En Route to Habitat Hotspots
Source: PLoS One. 2012 Feb 20;7(2):e31290. doi: 10.1371/journal.pone.0031290 (PMC3282722; doi:10.1371/journal.pone.0031290)

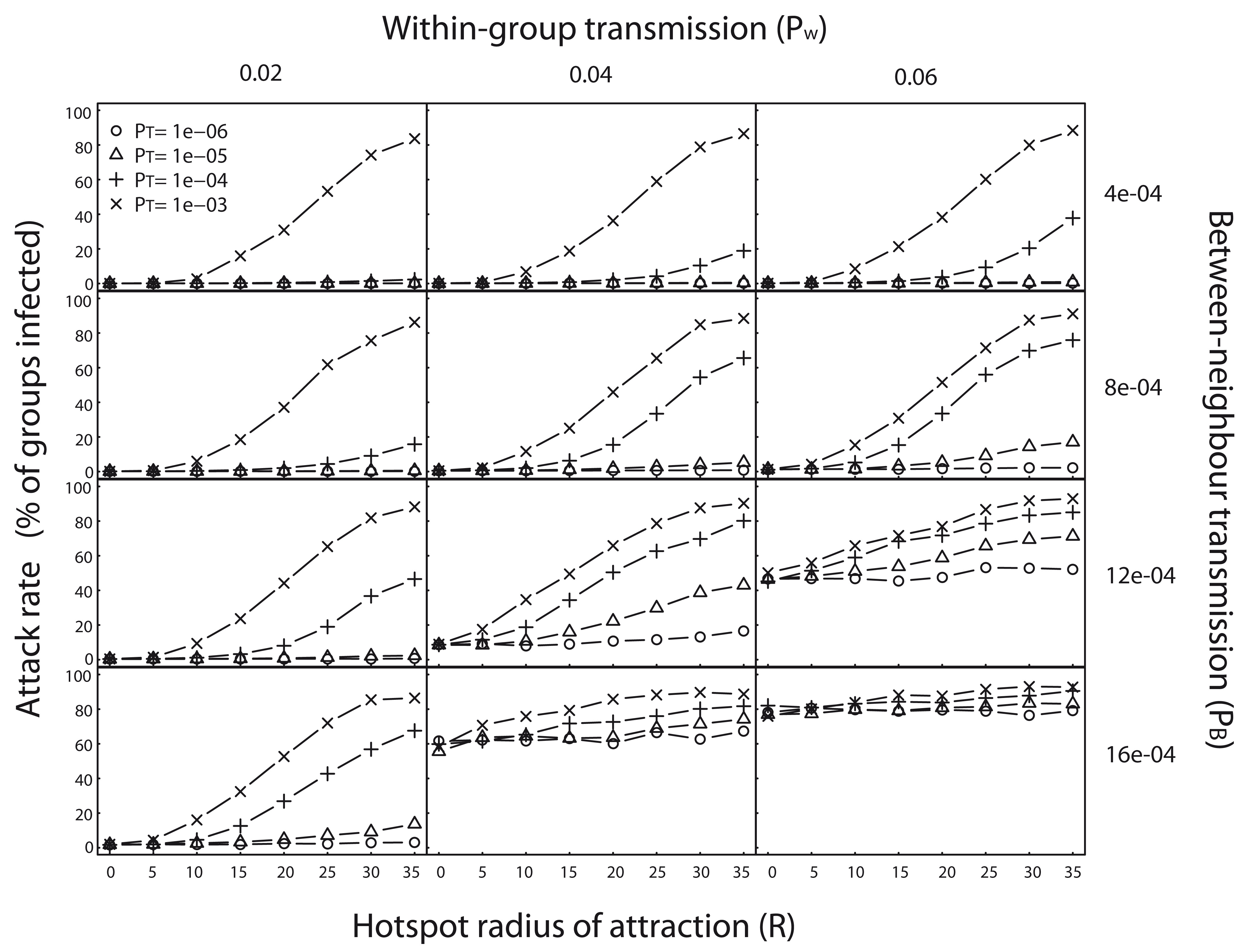

Supplement: Figure S1 — Influence of multiple model parameters on attack rate, when infected groups travel (Sick-travel model). The fraction of groups infected increases with the hotspot radius of attraction, but varies with the traveler-resident transmission probability PT (four lines in each graph), within-group transmission probability Pw (three different columns of graphs), and between-neighbour transmission probability PB (four different rows of graphs). Each value is based on 1000 simulations in which disease was introduced randomly in one of the eigth groups adjacent to the hotspot. (TIF) [file pone.0031290.s001.tif]

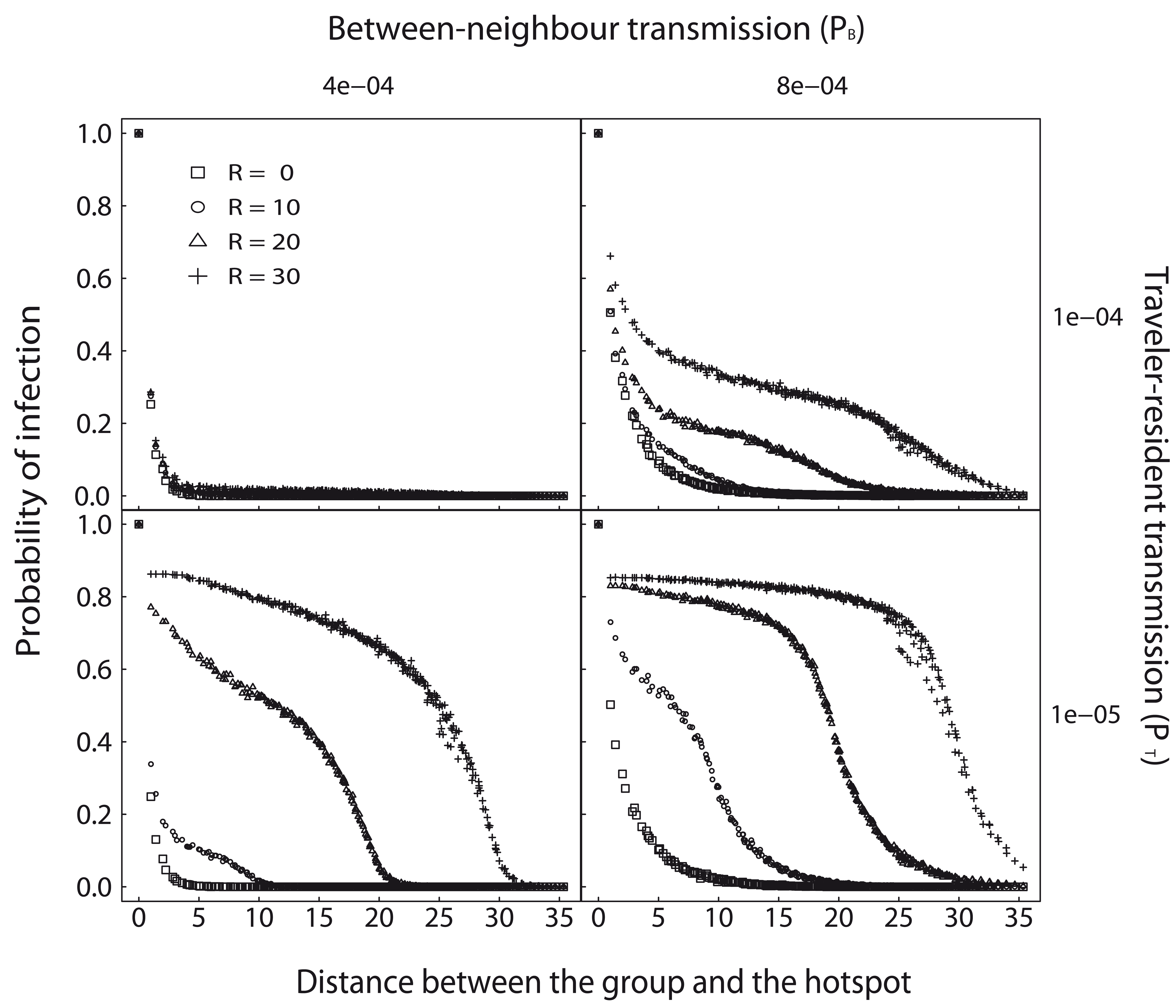

Supplement: Figure S2 — Group's probability of infection in relation to the distance to the hotspot, predicted by the Sick-travel model. The relationship is presented for different values of the hotspot radius of attraction (R). Each graph represents a combination of the between-neighbour (PB) and the traveler-resident (PT) transmission. The disease was introduced randomly in one of the eight groups adjacent to the hotspot. For all simulations, Pw = 0.06. (TIF) [file pone.0031290.s002.tif]

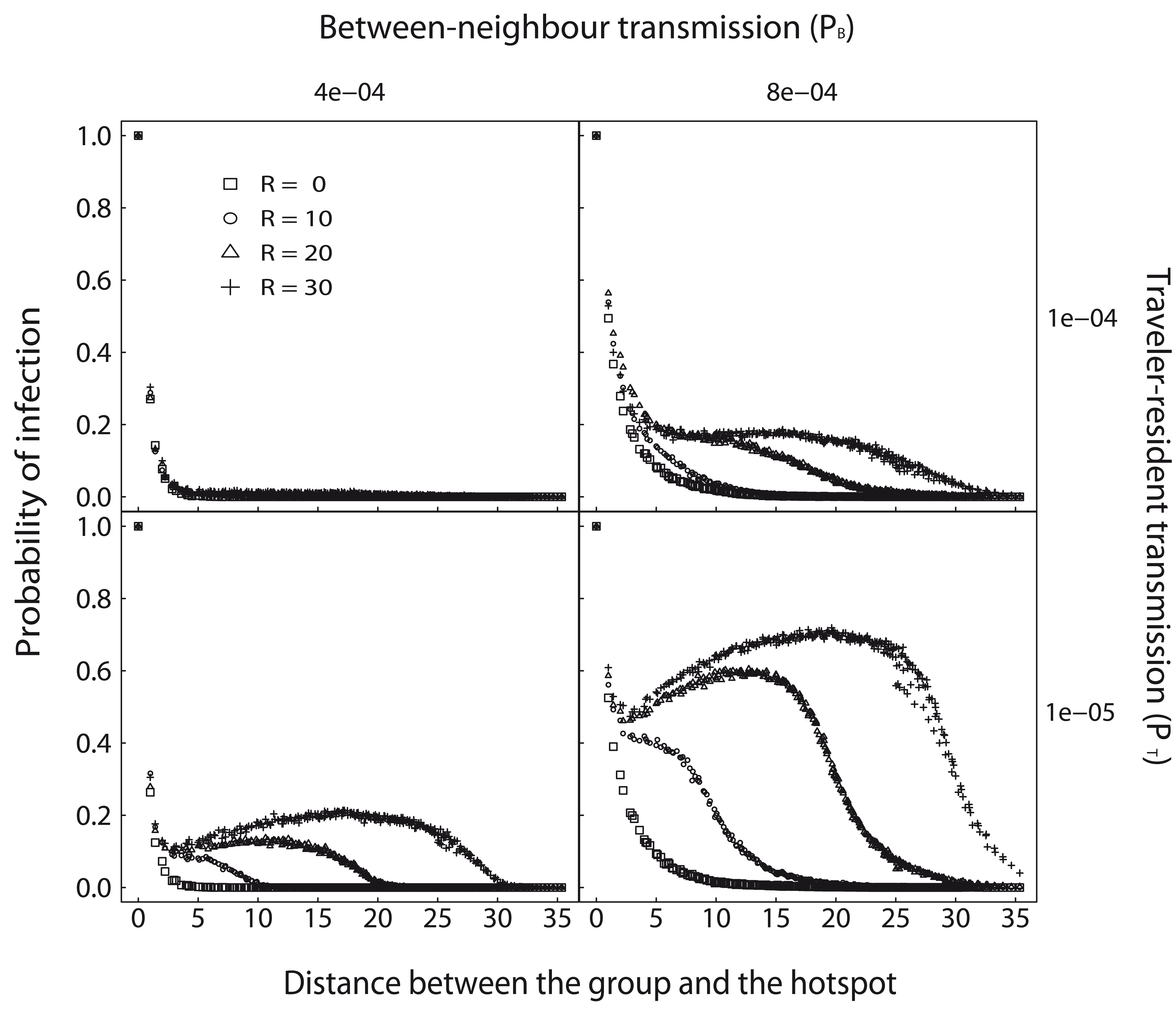

Supplement: Figure S3 — Group's probability of infection in relation to the distance to the hotspot, predicted by the Sick-stay model. The relationship is presented for different values of the hotspot radius of attraction (R). Each graph represents a combination of the between-neighbour (PB) and the traveler-resident (PT) transmission. The disease was introduced randomly in one of the eight groups adjacent to the hotspot. For all simulations, Pw = 0.06. (TIF) [file pone.0031290.s003.tif]

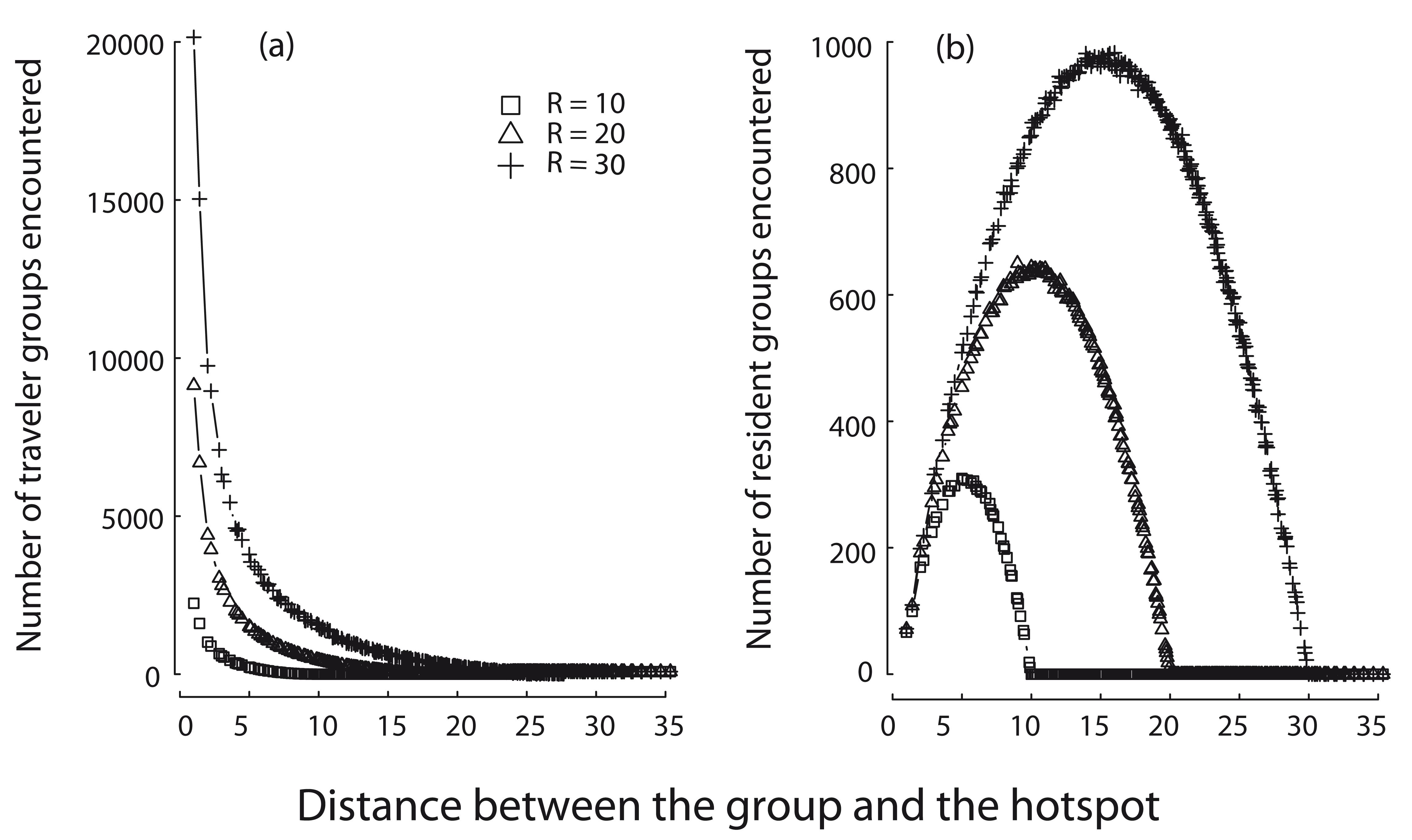

Supplement: Figure S4 — Traveler-resident contact patterns. Each graph shows the relationship between the distance of a group from the hotspot and (a) the number of other groups that travel through its territory when travelling to and from the hotspot, (b) the number of resident groups it encounters when travelling to and from the hotspot. Values are based on encounters occurring during 100 time steps, in the absence of disease transmission. (TIF) [file pone.0031290.s004.tif]
